# Clustering and drivers of symptoms observed at week six after antidepressant treatment in depressed outpatients

**DOI:** 10.1192/j.eurpsy.2024.1801

**Published:** 2025-01-17

**Authors:** Michel Danon, Daphnée Poupon, Philippe Courtet, Philip Gorwood

**Affiliations:** 1 Clinique des Maladies Mentales et de l’Encéphale, GHU Paris Psychiatrie et Neurosciences, Paris, France; 2 Université Paris Cité, Institute of Psychiatry and Neuroscience of Paris (IPNP), INSERM U1266, Paris, France; 3 Department of Emergency Psychiatry and Post-Acute Care, CHU Montpellier, France; 4 IGF, Hôpital La Colombière, University of Montpellier, Montpellier, France

**Keywords:** anxiety, cognitive, depression, emotional, residual symptoms

## Abstract

**Background:**

Depressive symptoms remaining after antidepressant treatment increase the risk of relapse and recurrence. We aimed to analyze the distribution and main drivers of remaining symptoms in patients with a major depressive episode.

**Methods:**

Two independent samples of 8,229 and 5,926 patients from two large naturalistic studies were retrospectively analyzed. DSM-IV criteria for major depressive episodes were assessed during two face-to-face visits with clinicians: before the prescription of a new antidepressant, and after 6 weeks of treatment. The Hospital Anxiety and Depression Scale (HADS) was used to assess baseline severity of anxiety and depression.

**Results:**

In both samples, two clusters of remaining symptoms were observed. The first cluster encompassed symptoms related to a negative emotional and cognitive bias and was specifically driven by the baseline severity of depression. The second cluster encompassed neurovegetative symptoms and was specifically driven by the baseline severity of anxiety.

**Conclusions:**

The baseline anxiety-depressive balance of patients could be considered to adapt the treatment, focusing on emotional and cognitive symptoms with patients with high baseline severity of depression, and neurovegetative symptoms with patients with high baseline anxiety severity.

## Introduction

The presence of residual depressive symptoms after weeks of antidepressant treatment has been the matter of research for a long time [1] as residual symptoms are detected in around 80% of patients after 8 weeks of selective serotonin reuptake inhibitor (SSRI) treatment [2], their number usually ranging from two to four [3,4]. Residual symptoms represent a real burden with functional impairment and poor life satisfaction [4]. Furthermore, residual symptoms are associated with a higher risk of relapses and/or recurrences [2,5]. Understanding the distribution and the main drivers of residual symptoms is therefore crucial, but these aspects have yet to be analyzed.

The definition of residual symptoms is not consensual. Some authors mention residual symptoms exclusively in subjects in remission [6,7], others only in subjects who have responded to antidepressant treatment [8,9], others again solely in subjects who have not achieved remission [1,10], and yet others in subjects who have received treatment without restriction regarding the degree of response [11]. Some authors have proposed a definition of residual symptoms as the “persistence of symptoms despite considerable clinical response to adequate therapy” [12]. However, it has been observed that the most common residual symptoms differed between responders (insomnia) and remitters (weight gain) [2,8,13]. To capture the maximum amount of information, consider all patients, and stick to the natural course of the disorder, the concept of *remaining symptoms* might be more appropriate. This concept also has the benefits of being able to focus on all symptoms that are present after a period of time (whatever its length) following a treatment process (whatever type of intervention), and of being independent of artificially defined response criteria and thresholds.

The most common remaining depressive symptoms are fatigue, anhedonia, and insomnia [14]. To treat a depressive episode, SSRIs are the most frequently used antidepressants as first-line treatment [15]. However, it appears that serotonergic modulation has little impact on these symptoms, unlike noradrenergic, dopaminergic, and histaminergic modulations [16]. Therefore, predicting remaining symptoms based on the initial clinical presentation is crucial for optimizing treatment selection.

Remaining depressive symptoms might be explained by neurocognitive abnormalities that tend to worsen with the number of episodes [17–19] and/or decreased efficacy of antidepressant treatments with therefore less extensive capacity to treat all depressive symptoms [20,21]. A long-term study on major depressive disorder showed that the risk of recurrence increased by 16% with each new episode, with a higher risk for a longer duration of recovery [22]. Aside from oxidative, nitrosative, and inflammatory stress, a frequently proposed explanation relies on smaller hippocampal volumes observed in patients with multiple episodes, with now some evidence of within-patient differences reinforcing the hypothesis of a cumulative impact of depressive episodes [23]. Such cumulative approaches tend to see remaining symptoms as the tail of a normal curve, with higher levels after each episode, but with symptoms tending to disappear with time. However, the remaining symptoms are not evenly distributed, for example, poor concentration, sleep disturbances and lack of energy/fatigue, and anxiety were more frequently observed [4,24,25].

In the present study, we aimed to analyze the distribution and the main drivers of remaining symptoms. To do so, we used the samples from two large naturalistic studies [19,26] that both assessed DSM-IV criteria before and after the prescription of an antidepressant treatment for a major depressive episode, with a similar follow-up of 6 weeks. We hypothesized that the remaining symptoms could be predicted by baseline severity, with more severe episodes leading to more remaining symptoms. We also hypothesized that the remaining symptoms were not evenly distributed, but instead organized in specific clusters driven by patients’ and/or disorder’s characteristics.

## Methods

### Participants

Two independent samples (sample 1 and sample 2) from two large naturalistic studies [19,26] were retrospectively analyzed. Participants were patients with a major depressive episode recruited from primary and psychiatric care clinical settings across France. After a complete description of the study to the subjects, written informed consent was obtained.

Broad inclusion and minimal exclusion criteria were used to ensure the representativeness of the sample. Patients were required to be diagnosed with a major depressive episode (at least five DSM-IV criteria) and about to start treatment with a new (or different) antidepressant, be over 18 years of age, speak fluent French, and possess a social security number. Non-inclusion criteria were: bipolar disorder, schizophrenia, primary substance misuse or primary organic disease, current treatment with an antipsychotic or a mood stabilizer, and pregnancy or lactation. All antidepressants (in accordance with the French equivalent of the Food and Drug Administration) were accepted to reflect usual clinical practice.

### Procedure

To recruit patients, two independent lists of 4,849 (sample 1) and 3,000 (sample 2) medical doctors were contacted by mail. They were invited to take part in a short-term follow-up protocol to which 3,375 (sample 1) and 2,088 (sample 2) agreed. They were asked to include, within a 3-month interval, consecutive patients with a major depressive episode according to the DSM-IV criteria for whom a new (or different) antidepressant had to be prescribed. A maximum number of five patients was requested to avoid center effects. Ultimately, 1,844 (sample 1) and 1,186 (sample 2) clinicians included at least one patient who was followed up with a 6-week delay between the two visits. Each participating investigator was contacted (usually by phone) at least twice: at the beginning of the protocol (to ensure that the protocol was clear) and at the close of trial entry (to verify the received data).

Patients were seen by the clinician during two face-to-face visits: at inclusion (baseline) and 6 weeks later.

During both visits, the clinician examined the nine DSM-IV criteria for major depressive episodes, namely depressed mood, diminished interest, appetite disturbance, sleep disturbance, psychomotor disturbance, fatigue, feelings of worthlessness, diminished ability to think or concentrate, and suicidal thoughts. At baseline, the presence of five or more symptoms was required for inclusion. The remaining symptoms were the DSM-IV symptoms that were still present at follow-up.

The Hospital Anxiety and Depression Scale (HADS) was completed at both visits. This 14-item self-questionnaire measures symptom severity through four-point Likert scales rated from 0 to 3 [27]. The HADS was chosen because of its rapidity and simplicity of rating. HADS anxiety and depression scores, each ranging from 0 to 21, with higher scores reflecting more severe symptoms, were analyzed separately to determine baseline severity.

In addition to DSM-IV symptoms and HADS scores, sociodemographic data, number of past depressive episodes, and length of current episode were collected at baseline. Any change of antidepressant, increase in the dosage, or addition of a benzodiazepine, were recorded at the second visit.

### Statistical analysis

Statistical analyses were performed using the SPSS software^®^ (IBM Corp., Released 2021, SPSS Statistics for Macintosh, Version 28.0, Armonk).

Pearson’s correlations were used to compare continuous variables. The *t*-tests were used to compare groups for quantitative data. Multiple linear regressions were used to analyze the role of binary or continuous independent variables to explain a continuous dependent variable. The normality of distribution was assessed graphically. A principal component analysis was performed to explore the structure of remaining symptoms and extract loading variables for each factor. Only factors with an eigenvalue >1 were retained. The varimax orthogonal rotation method was used to maximize the independence of the factors. The Anderson–Rubin scoring method was used so that the factor-loading variables were not correlated.

## Results

A total of 8,229 (sample 1) and 5,926 (sample 2) patients were included in the study. Among them, 7,809 (sample 1) and 5,473 (sample 2) patients had complete data regarding the DSM criteria for depression at the second visit. Apart from the fact that sample 1 was larger and with a higher proportion of women (70% vs. 63%) than sample 2, mean (SD) values were similar in both samples (Table 1).

**Table 1.** Description of the two samples

	Sample 1	Sample 2
	*n* = 8229	*n* = 5926
Gender, No (%) females	5796 (70.4)	3748 (63.2)
Age	48.02 (14.09)	47.24 (14.04)
Number of past episodes	0.97 (1.42)	1.12 (1.71)
Length of the current episode	8.37 (10.84)	7.66 (12.46)
Number of DSM symptoms at baseline	6.67 (1.14)	7.13 (1.20)
HADS depression score at baseline	14.62 (3.24)	14.97 (3.77)
HADS anxiety score at baseline	14.12 (3.01)	14.25 (3.46)

*DSM*, Diagnostic and Statistical Manual of Mental Disorders; *HADS*, Hospital Anxiety and Depression Scale.

Unless “No (%)” is stated, data are described as Mean (SD).

At week 6, the three most prevalent symptoms were found in the same order in both samples, namely (sample 1 and sample 2): fatigue (65.3% and 72.1%), diminished ability to think (42.6% and 50.5%) and diminished interest (39.8% and 49.9%). The two least frequent symptoms were also the same in both samples: appetite disturbance (17.9% and 22.0%) and suicidal thoughts (4.6% and 12.6%). The fourth and fifth most prevalent symptoms had their order reversed between the two samples: sleep disturbance (35.8% and 37.9%) and depressed mood (34.4% and 42.4%). The same applied to the sixth and seventh most frequent symptoms: psychomotor disturbance (26.0% and 32.9%) and worthlessness (25.7% and 36.5%). The average number of remaining symptoms was 2.88 (SD = 1.96) in sample 1 and 3.51 (SD = 2.29) in sample 2.

The number of remaining symptoms in sample 1 correlated with older age, more depressive episodes, longer duration of the current episode, higher baseline severity of both depression and anxiety, type of antidepressant treatment, and female gender (all *p*-values <.001). These results were replicated in sample 2, apart from the role of gender which was not significant (*t* = 1.126, *p* = .260). The number of remaining symptoms was not significantly associated with marital status (*t* = .410, *p* = .682) or professional status (*t* = 1.458, *p* = .145). To control for collinearity, a regression analysis was performed and confirmed the role of older age, female gender, more depressive episodes, longer duration of the current episode, type of antidepressant treatment, and higher baseline severity of depression and anxiety (*p* < .009) in sample 1. In sample 2, the role of age (*t* = 1.429, *p* = .153) and gender (*t* = −.073, *p* = .942) was not confirmed but other factors came out as significant (*p* < .050).

**Figure 1.**
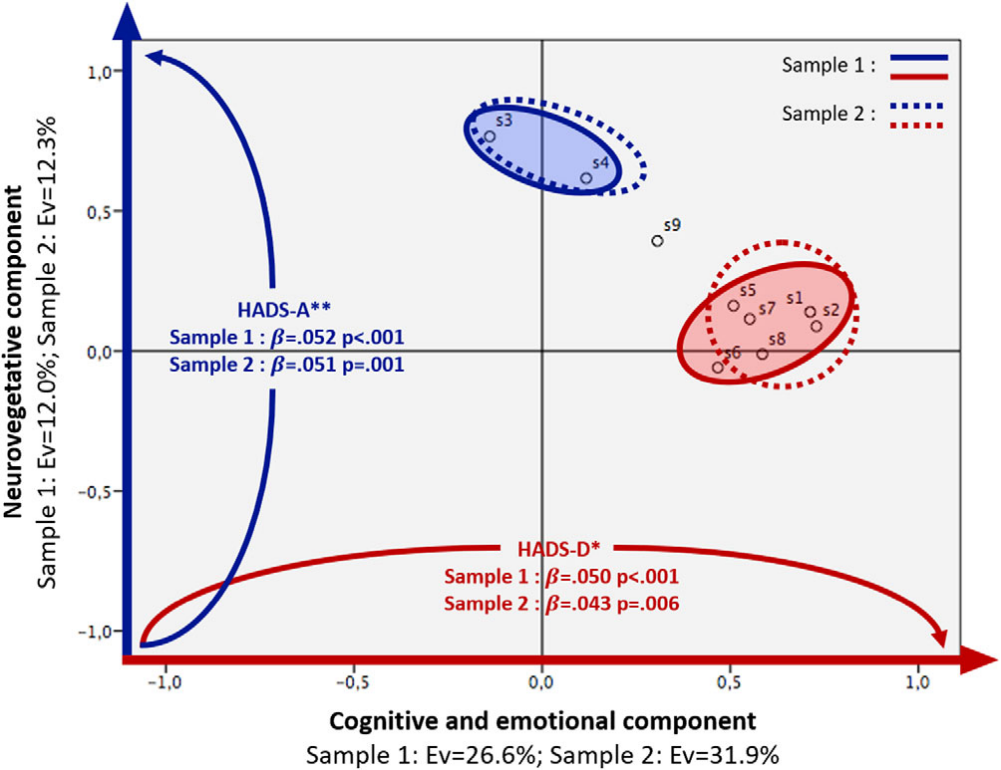
**Schematic representation of a PCA of remaining depressive symptoms after 6 weeks of treatment** in two independent samples (Sample 1, *n* = 7,809 and Sample 2, *n* = 5,473) and the role of depressive versus anxious symptoms as drivers of each cluster. **β**: standardized regression coefficients; **DSM**: Diagnostic and Statistical Manual of Mental Disorders; **Ev**: explained variance; **HADS**: Hospital Anxiety and Depression Scale; HADS-D*: prediction of factor 1 loading score (PCA) by depression baseline score (HADS) adjusted for anxiety baseline score (HADS), age, gender, length of current episode, number of past episodes and type of antidepressant treatment; HADS-A**: prediction of factor 2 loading score (PCA) by anxiety baseline score (HADS) adjusted for depression baseline score (HADS), age, gender, length of current episode, number of past episodes and type of antidepressant treatment; **p**: *p* value; **PCA**: Principal Component Analysis; **s1**: depressed mood (DSM-IV); **s2**: diminished interest (DSM-IV); **s3**: appetite disturbance (DSM-IV); **s4**: sleep disturbances (DSM-IV); **s5**: psychomotor disturbance (DSM-IV); **s6**: fatigue (DSM-IV); **s7**: worthlessness (DSM-IV); **s8**: diminished ability to think (DSM-IV); **s9**: suicidal thoughts (DSM-IV).

We then looked at how the remaining symptoms were distributed at week 6 with a principal component analysis. In sample 1, it detected two clusters with an eigenvalue above 1, explaining 26.6% and 12.0% of the global variance. Two clusters were also observed in sample 2, with 31.9% and 12.3% of explained variance. These two clusters encompassed the same symptoms in both samples, with depressed mood (symptom 1), diminished interest (symptom 2), psychomotor disturbance (symptom 5), fatigue (symptom 6), worthlessness (symptom 7), diminished ability to think (symptom 8), and suicidal thoughts (symptom 9) in the first cluster that we entitled *cognitive and emotional*, while appetite and sleep disturbances (symptoms 3 and 4) constituted the second cluster that we entitled *neurovegetative* (Supplementary information S1 and S2). The only change after varimax rotation was that symptom 9 did not belong to any of the two clusters anymore (Table 2 and Figure 1).

**Table 2.** Clustering (PCA) of remaining symptoms in both samples (varimax)

	Sample 1		Sample 2	
	Component 1	Component 2	Component 1	Component 2
Symptom 1	.712	.139	.692	.292
Symptom 2	.729	.088	.734	.204
Symptom 3	−.139	.766	−.107	.797
Symptom 4	.117	.616	.166	.634
Symptom 5	.508	.161	.510	.268
Symptom 6	.467	−.059	.578	−.049
Symptom 7	.552	.114	.645	.058
Symptom 8	.585	−.011	.622	−.032
Symptom 9	.306	.393	.379	.373

**Symptom 1**: depressed mood (DSM-IV); **Symptom 2**: diminished interest (DSM-IV); **Symptom 3**: appetite disturbance (DSM-IV); **Symptom 4**: sleep disturbances (DSM-IV); **Symptom 5**: psychomotor disturbance (DSM-IV); **Symptom 6**: fatigue (DSM-IV); **Symptom 7**: worthlessness (DSM-IV); **Symptom 8**: diminished ability to think (DSM-IV); **Symptom 9**: suicidal thoughts (DSM-IV)

Looking at the drivers of these two clusters of remaining symptoms, we found that the *cognitive and emotional* cluster was non-specifically explained in sample 1 by all factors previously associated with the number of remaining symptoms, while the *neurovegetative* cluster was only driven by number of past depressive episodes, length of the current one and baseline anxiety symptoms, with a smaller effect of baseline depressive severity (*p* = .044). Sample 2 showed a relatively equivalent distribution of drivers, apart from gender and length of the current episode that did not correlate with cluster 1. Once again, as collinearity can be an issue, we assessed these drivers with regression analyses and found in both samples that the baseline severity of depression was specific to cluster 1, while the baseline severity of anxiety was specific to cluster 2 (Table 3 and Figure 1). The effect of types of antidepressant treatment was also specific to cluster 1 (for Factor 1: *F* = 7.98, *p* < .001 in sample 1 and *F* = 3.06, *p* = .027 in sample 2; for Factor 2: *F* = 1.42, *p* = .235 in sample 1 and *F* = 0.65, *p* = .582 in sample 2).

**Table 3.** Drivers of the two clusters (multiple linear regressions)

	Sample 1					Sample 2				
	Dependent variable: Factor 1					Dependent variable: Factor 1				
Independent variables	*B*	*σ*	*β*	*t*	*p*	*B*	*σ*	*β*	*t*	*p*
Intercept	−0.663	0.075		−8.817	< .001	−0.497	0.090		−5.500	< .001
Gender	−0.087	0.025	−0.087	−3.518	< .001	0.012	0.030	0.012	0.412	.681
Age	0.007	0.001	0.092	8.174	< .001	0.003	0.001	0.036	2.497	.013
Number of past episodes	0.036	0.010	0.050	3.808	< .001	0.043	0.009	0.070	4.873	< .001
Length of current episode	0.002	<.001	0.072	5.438	< .001	0.004	0.001	0.041	2.883	.004
HADS depression score at baseline	0.016	0.004	0.050	4.153	< .001	0.012	0.004	0.043	2.768	.006
HADS anxiety score at baseline	0.005	0.004	0.014	1.147	.251	0.008	0.005	0.028	1.773	.076
Antidepressant										
SSRI – Other	−0.021	0.097	−0.021	−0.214	.830	−0.230	0.109	−0.230	−2.113	.035
SNRI – Other	−0.161	0.121	−0.161	−1.324	.186	−0.016	0.117	−0.016	−0.139	.889
Atypical tricyclic – Other	−0.236	0.082	−0.236	−2.865	.004	−0.124	0.099	−0.124	−1.253	.210

	Dependent variable: Factor 2					Dependent variable: Factor 2				
Independent variables	*B*	*σ*	*β*	*t*	*p*	*B*	*σ*	*β*	*t*	*p*
Intercept	−0.267	0.076		−3.511	< .001	−0.230	0.091		−2.514	.012
Gender	−0.014	0.025	−0.014	−0.552	.581	−0.006	0.030	−0.006	−0.184	.854
Age	<.001	0.001	−0.007	−0.568	.570	−0.002	0.001	−0.028	−1.900	.057
Number of past episodes	0.018	0.010	0.024	1.822	.068	0.039	0.009	0.064	4.436	< .001
Length of current episode	0.002	<.001	0.053	4.001	< .001	0.004	0.001	0.042	2.934	.003
HADS depression score at baseline	<.001	0.004	<.001	0.035	.972	0.003	0.004	0.012	0.748	.455
HADS anxiety score at baseline	0.017	0.004	0.052	4.262	< .001	0.015	0.005	0.051	3.218	.001
Antidepressant										
SSRI – Other	−0.064	0.098	−0.064	−0.650	.516	−0.112	0.110	−0.111	−1.016	.309
SNRI – Other	−0.131	0.123	−0.131	−1.069	.285	−0.137	0.118	−0.136	−1.159	.247
Atypical tricyclic – Other	−0.136	0.083	−0.136	−1.627	.104	−0.074	0.100	−0.074	−0.746	.456

***β**: standardized regression coefficients; **σ**: standard deviation; **B**: regression coefficients; **DSM**: Diagnostic and Statistical Manual of Mental Disorders; **Factor 1**: factor 1 loading variable resulting from PCA by the Anderson-Rubin scoring method; **Factor 2**: factor 2 loading variable resulting from PCA by the Anderson-Rubin scoring method; **HADS**: Hospital Anxiety and Depression Scale; **p**: p value; **PCA**: Principal Component Analysis; **SNRI**: serotonin–norepinephrine reuptake inhibitors; **SSRI**: selective serotonin reuptake inhibitor; **t**: t value*

As baseline depressive and anxiety scores seemed to act differently to predict clusters of remaining symptoms, we assessed how cluster 1 versus cluster 2 symptoms after 6 weeks of treatment could predict baseline depressive versus anxiety severity by performing two regression analyses. Controlling for baseline anxiety severity, factor 1 score was indeed able to predict baseline depressive severity (*p* < .003) in both samples, but not factor 2 score (*p* > .444), and controlling for baseline depressive severity, factor 2 score was able to predict baseline anxiety severity (*p* < .001), but not factor 1 score (*p* > .060) in both samples (Supplementary information S3).

## Discussion

By studying two samples of 8,229 and 5,926 patients, we were able to demonstrate the existence of two clusters in the distribution of remaining symptoms. The first cluster encompassed negative bias symptoms and was specifically driven by the initial severity of depression, while the second cluster encompassed neurovegetative symptoms and was specifically driven by the initial severity of anxiety.

Studies focusing on the structure of depressive symptoms have employed various clustering methods such as PCA [28–32], factor analyses [9,32–38], latent class analyses [32], and hierarchical clustering [39–44]. Other authors have employed network models such as the least absolute shrinkage and selection operator [11,45–48] and dynamic time warping [39,41,42,44]. These clustering studies have focused on different stages of depression: some involved patients currently experiencing depression [28,29,32,33,35–38,40], while others investigated symptom evolution during antidepressant treatment [31,39,41–44], and still others examined symptoms persisting after receiving antidepressant treatment [9,11,48]. These clustering studies employed various depression scales such as the Patient Health Questionnaire-9 (corresponding to DSM-IV symptoms) [32,33,35,36], the Hamilton Rating Scale for Depression [32,37,40,42], the Inventory of Depressive Symptomatology [29,32,41,44], the Montgomery–Åsberg Depression Rating Scale [31,32], or the Beck Depression Inventory [30,43]. Other studies incorporated additional data into clustering methods such as items from the General Anxiety Disorder-7 [28,38], functional imaging [43], or biology [30]. These clustering studies have identified between two [29,33,35,36,41,43] and five clusters [28,42]. Similar to our study, sadness and anhedonia tend to load on the same factors, as do sleep disturbance and appetite disturbance [28,32]. Several studies, like ours, have identified a neurovegetative or somatic cluster incorporating sleep and appetite disturbances [29–31,33,35,36,38,39]. The majority of studies, like ours, have found a core/emotional/affective/cognitive cluster [29,31,33,35–44]. Sadness and anhedonia are consistently found in the central symptoms of network models [11,45,47]. It is interesting to note that remitted subjects exhibit the same symptom structure as depressed individuals, resembling a depressive scar [48].

In our PCA, symptom 9 (suicidal ideation) belonged to the cognitive and emotional cluster before rotation, but the orthogonal Varimax rotation removed this symptom from all clusters. It is important to note that this type of rotation, commonly used in many studies, produces the most independent clusters possible [29,32]. This characteristic is suitable for our objective, as it is reasonable to assume that the more independent these clusters are, the more independent their predictors will be. Some authors find that symptom 9 belongs to the core/emotional/affective/cognitive cluster [33,35,36,38,43,44], others to the neurovegetative/somatic cluster [41], and still others observe that symptom 9 belongs to a psychomotor cluster (agitation/slowing) [29,39,40,42].

Our results suggest a strict dichotomy between the two clusters; however, this might not fully reflect reality. First, as seen in Figure 1, suicidal ideation does not segregate between the two clusters. Secondly, the varimax orthogonal rotation and the Anderson–Rubin scoring method maximize the independence of the factors. Consequently, there was no significant correlation between the factor loading variables, which is more a result of these statistical methods than a reflection of the more complex clinical reality. Similarly, the previously described anxiety-depression balance does not imply a dichotomy between anxiety and depression symptoms. In fact, research on these entities, which are distinct according to the DSM, has highlighted their frequent co-occurrence, shared vulnerability factors, and the common effectiveness of antidepressants in treating both disorders [49].

In depression, a negative emotional and cognitive bias colors the processing of information and rewards, contributing to symptoms of sadness and worthlessness (symptoms 1 and 7), anhedonia and reduced sensitivity to reward (symptom 2) [50], lack of motivation and hypersensitivity to effort (symptom 6) [51], and deficits in disengagement from self-centered negative rumination (symptoms 5 and 8) [17,52,53], giving sense to the symptoms merged in cluster 1 that we entitled *cognitive and emotional.* Monitoring these symptoms in cases of high initial depressive severity therefore appears to be useful, particularly in elderly patients. Cognitive Behavioral Therapy for Depression (CBT-D) according to Beck’s model could be a treatment of choice in this case, directly addressing the expectative top-down negative bias in information processing by countering selective abstraction processes and shifting away from negative thoughts (symptoms 1 and 7) [54,55]. Symptom 6 is a frequent residual symptom, highly predictive of the inability to achieve remission [56]. In this scenario, augmenting treatment through noradrenergic or dopaminergic modulation may be of interest. While some studies have shown the positive effects of these augmentations on fatigue, the evidence remains insufficient, necessitating further studies [56]. Additionally, it is important to note the effect of serotonergic stimulation on reducing effort sensitivity, a component of fatigue, whereas dopaminergic stimulation appears to improve only reward sensitivity, thus favoring combined approaches [51,57,58]. Nevertheless, modulation of the dopaminergic system by serotonin and the use of noradrenaline reuptake transporters by dopamine nuanced previous observations, making a potential monoaminergic cleavage porous and prompting humility regarding overly simplistic conceptualizations [59]. Remaining symptoms 5 and 8 (psychomotor disturbance and diminished ability to think) may be explained by a deficit in disengagement from self-centered negative rumination, which is associated with an alteration in the dynamics between the Default Mode Network (DMN) and the Task Positive Network (TPN) [17,52,53]. These particularities are predictive of depressive relapse. Mindfulness-Based Cognitive Therapy (MBCT), recommended for the prevention of depressive relapse and effective on residual depressive symptoms, is an interesting strategy for this situation as it could restore this network dynamic and reduce negative rumination [60–64].

Sleep and appetite disturbances are neurovegetative symptoms of depression [2]. According to our findings, particular attention should be paid to these symptoms in patients with high initial anxiety severity. Monitoring sleep during the treatment of depression is particularly important as its early improvement is associated with higher chances of achieving remission [65]. The most common type of residual insomnia is mid-nocturnal insomnia [12]. Sleep disturbances can be side effects of certain antidepressants, which may then need to be adjusted [12]. Cognitive Behavioral Therapy for Insomnia (CBT-I) is a treatment of choice for residual insomnia, providing rapid and lasting effects in just six to eight sessions [12]. Other strategies appear promising, such as MBCT, Behavioral Activation (BA), and some anti-epileptic drugs [12]. Just like sleep disturbances, appetite, and weight disturbances can be side effects of certain antidepressants, which may then need adjustment to avoid deleterious metabolic consequences.

A limitation of this study is a lack of precision in the description of symptoms. Symptoms 3, 4, and 5 of depression according to the DSM-IV are alterations that can go in two opposite directions: anorexia or hyperphagia, hypersomnia or insomnia, and retardation or agitation. Our study rates the alteration but does not make the distinction. This distinction is important because, for example, insomnia is associated with symptoms such as irritability, psychomotor agitation, and anxiety [66]. The lack of distinction could have a consequence on the neurovegetative cluster, which could be reflective of a failure to treat atypical depression, a particular entity characterized by symptoms of hyperphagia and hypersomnia. While 85% of patients with depression exhibit insomnia symptoms, 50% present with hypersomnia symptoms, and 15%–40% display atypical features [67,68]. Atypical depression has been associated with the female sex, more severe symptoms, a greater number of recurrences, more anxious comorbidities, more bipolar disorders, less physical activity, more isolation, and more metabolic, cardiovascular, and inflammatory alterations [69–72]. Some studies have demonstrated the particular efficacy of Monoamine Oxidase Inhibitors (MAOIs), physical exercise, and CBT-D in the treatment of atypical depression [72,73]. Some authors advocate for systematic screening for bipolar disorder in patients presenting with atypical depression and the use of drugs with antidepressant and mood-stabilizing properties as appropriate [72]. Another level of complexity to consider is the fact that the co-occurrence of insomnia and hypersomnia in depression is around 30%, and this subgroup shows higher rates of bipolar disorder [67]. Another limitation is that, at 6 months of antidepressant prescription, ~46% of patients are no longer adherent [74]. We do not have data on treatment adherence in our study, and not taking into account this potential confounding factor may introduce statistical noise in the interpretation of remaining symptoms. Nevertheless, the follow-up duration here is only 6 weeks, which reduces (but does not suppress) the likelihood of non-adherence [75]. Another limitation is the lack of information regarding the comorbidities. However, the large number of patients and the replication of results across two independent samples mitigate the impact of these biases. Also, the concept of early remaining symptoms (at 6 weeks) could seem odd. Nevertheless, other studies focusing on the structure of depressive symptoms have looked at symptoms after 4–8 weeks of treatment [31,34,43]. This early assessment appears logical since it allows quick feedback on efficacy with a larger margin of actions. Furthermore, while this short follow-up does not allow us to directly assess late responses and long-term remissions, it does have the benefit of limiting dropouts and associated biases. Finally, guidelines require only 4 weeks of treatment to diagnose treatment-resistant depression, and our study is in line with this early intervention approach, justified by the poor prognosis of long-lasting depression and residual symptoms [76].

In conclusion, after 6 weeks of antidepressant treatment, we identified two clusters of remaining symptoms: a cluster of symptoms having in common a negative emotional and cognitive bias, specifically driven by the initial depressive severity, and an independent neurovegetative cluster, specifically driven by baseline anxiety severity. In cases of high baseline depressive versus baseline anxiety severity, it appears useful to focus the attention on emotional and cognitive symptoms versus on neurovegetative symptoms. Specific treatment as an add-on or associated psychotherapy could then be proposed early, targeting these specific sets of symptoms that are more likely to remain.

## Supporting information

Danon et al. supplementary material Danon et al. supplementary material

## Data Availability

Data are available from the corresponding author upon reasonable request.

## Supplementary material

The supplementary material for this article can be found at http://doi.org/10.1192/j.eurpsy.2024.1801.
