# Supplementary material for: Clustering and drivers of symptoms observed at week six after antidepressant treatment in depressed outpatients
Source: Eur Psychiatry. 2025 Jan 17;67(1):e85. doi: 10.1192/j.eurpsy.2024.1801 (PMC11795446; doi:10.1192/j.eurpsy.2024.1801)
Supplement: Danon et al. supplementary material [file S0924933824018017sup001.docx]

**Supplementary information**

| **S1: Clustering (PCA) of remaining symptoms in both samples (without rotation)** | | | | | | | | | |
| --- | --- | --- | --- | --- | --- | --- | --- | --- | --- |
|  |  |  |  |  |  |  |  |  |  |
|  |  | Sample 1 | | |  | Sample 2 | | |  |
|  |  |  |  |  |  |  |  |  |  |
|  |  | Component 1 |  | Component 2 |  | Component 1 |  | Component 2 |  |
|  |  |  |  |  |  |  |  |  |  |
| Symptom 1 |  | .722 |  | -.070 |  | .751 |  | -.005 |  |
| Symptom 2 |  | .723 |  | -.124 |  | .755 |  | -.102 |  |
| Symptom 3 |  | .085 |  | .774 |  | .216 |  | .775 |  |
| Symptom 4 |  | .288 |  | .557 |  | .403 |  | .517 |  |
| Symptom 5 |  | .533 |  | .009 |  | .574 |  | .046 |  |
| Symptom 6 |  | .430 |  | -.190 |  | .512 |  | -.273 |  |
| Symptom 7 |  | .561 |  | -.048 |  | .615 |  | -.201 |  |
| Symptom 8 |  | .557 |  | -.178 |  | .559 |  | -.274 |  |
| Symptom 9 |  | .406 |  | .289 |  | .495 |  | .194 |  |
|  |  |  |  |  |  |  |  |  |  |
| ***Symptom 1****: depressed mood (DSM-IV);* ***Symptom 2****: diminished interest (DSM-IV);* ***Symptom 3****: appetite disturbance (DSM-IV);* ***Symptom 4****: sleep disturbances (DSM-IV);* ***Symptom 5****: psychomotor disturbance (DSM-IV);* ***Symptom 6****: fatigue (DSM-IV);* ***Symptom 7****: worthlessness (DSM-IV);* ***Symptom 8****: diminished ability to think (DSM-IV);* ***Symptom 9****: suicidal thoughts (DSM-IV)* | | | | | | | | | |

**S2: Scree plots (principal component analysis)**


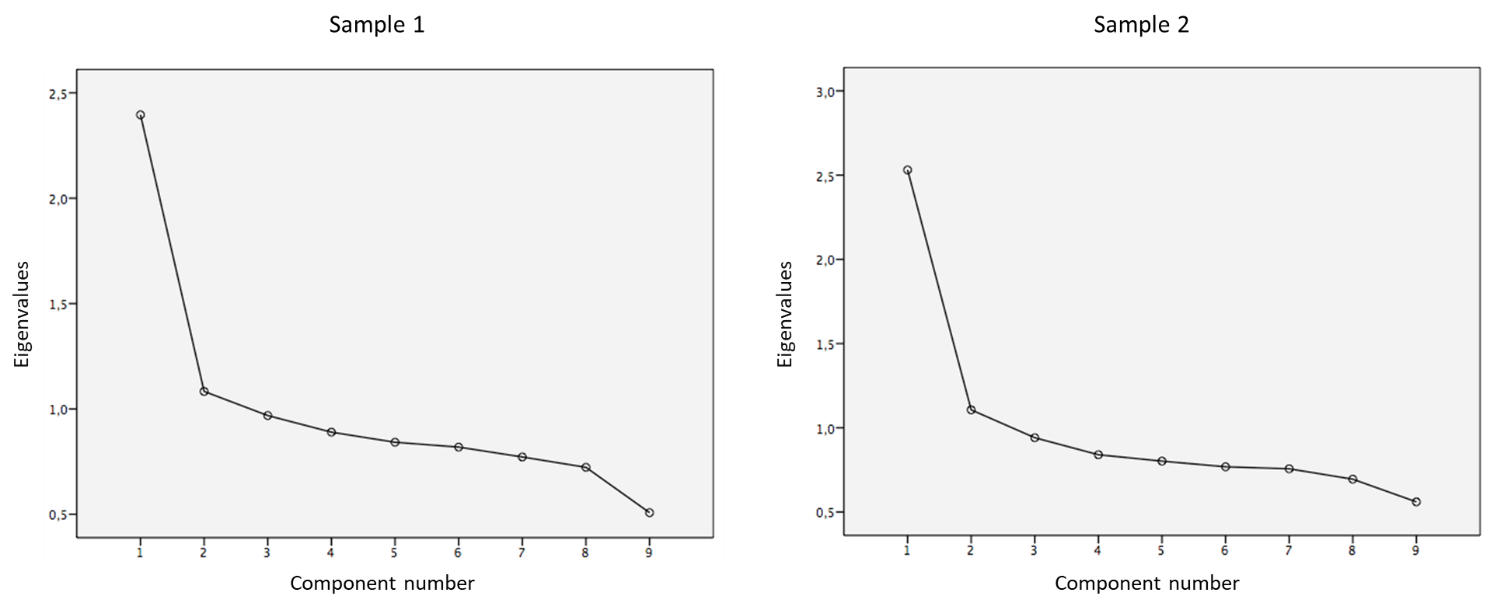


| **S3: Prediction of baseline anxiety and depression (HADS) according to cluster belonging (PCA)** | | | | | | | | | | | | |
| --- | --- | --- | --- | --- | --- | --- | --- | --- | --- | --- | --- | --- |
|  |  |  |  |  |  |  |  |  |  |  |  |  |
|  |  | Sample 1 | | | | |  | Sample 2 | | | | |
|  |  |  |  |  |  |  |  |  |  |  |  |  |
|  |  | Dependent variable: HADS-D | | | | |  | Dependent variable: HADS-D | | | | |
|  |  |  |  |  |  |  |  |  |  |  |  |  |
| Independent variables |  | B | σ | β | t | p |  | B | σ | β | t | p |
|  |  |  |  |  |  |  |  |  |  |  |  |  |
| Intercept |  | 9.054 | 0.163 |  | 55.406 | < .001 |  | 8.988 | 0.202 |  | 44.490 | < .001 |
| Factor 1 |  | 0.174 | 0.034 | 0.054 | 5.112 | < .001 |  | 0.140 | 0.048 | 0.037 | 2.944 | .003 |
| Factor 2 |  | 0.005 | 0.034 | 0.001 | 0.134 | .894 |  | 0.036 | 0.047 | 0.010 | 0.766 | .444 |
| HADS-A |  | 0.396 | 0.011 | 0.369 | 35.040 | < .001 |  | 0.421 | 0.014 | 0.388 | 30.621 | < .001 |
|  |  |  |  |  |  |  |  |  |  |  |  |  |
|  |  | Dependent variable: HADS-A | | | | |  | Dependent variable: HADS-A | | | | |
|  |  |  |  |  |  |  |  |  |  |  |  |  |
| Independent variables |  | B | σ | β | t | p |  | B | σ | β | t | p |
|  |  |  |  |  |  |  |  |  |  |  |  |  |
| Intercept |  | 9.104 | 0.147 |  | 62.010 | < .001 |  | 8.905 | 0.181 |  | 49.320 | < .001 |
| Factor 1 |  | 0.045 | 0.032 | 0.015 | 1.420 | .155 |  | 0.083 | 0.044 | 0.024 | 1.880 | .060 |
| Factor 2 |  | 0.149 | 0.032 | 0.050 | 4.740 | < .001 |  | 0.163 | 0.044 | 0.047 | 3.730 | < .001 |
| HADS-D |  | 0.343 | 0.010 | 0.369 | 35.040 | < .001 |  | 0.358 | 0.012 | 0.388 | 30.620 | < .001 |
|  |  |  |  |  |  |  |  |  |  |  |  |  |
| ***β****: standardized regression coefficients;* ***σ****: standard deviation;* ***B****: regression coefficients;* ***DSM****: Diagnostic and Statistical Manual of Mental Disorders;* ***Factor 1****: factor 1 loading variable resulting from PCA by the Anderson-Rubin scoring method;* ***Factor 2****: factor 2 loading variable resulting from PCA by the Anderson-Rubin scoring method;* ***HADS****: Hospital Anxiety and Depression Scale;* ***HADS-A****: HADS anxiety score at baseline;* ***HADS-D****: HADS depression score at baseline;* ***p****: p value;* ***PCA****: Principal Component Analysis;* ***t****: t value* | | | | | | | | | | | | |
